# 共表达IL-7/CCL19的新抗原反应性T细胞对小鼠肺癌的抗肿瘤研究

**DOI:** 10.3779/j.issn.1009-3419.2024.106.18

**Published:** 2024-07-20

**Authors:** Di WU, Chenhui LI, Yan WANG, Zhengqiang HE, Chang’e JIN, Min GUO, Rongchang CHEN, Chengzhi ZHOU

**Affiliations:** ^1^518020 深圳，暨南大学第二临床医学院（深圳市人民医院）深圳市呼吸疾病研究所肺重症医学科，南方科技大学第一附属医院（深圳市人民医院）; ^1^Department of Pulmonary and Critical Care Medicine, Shenzhen Institute of Respiratory Diseases, The Second Clinical Medical College, Jinan University (Shenzhen People’s Hospital), The First Affiliated Hospital of Southern University of Science and Technology (Shenzhen People’s Hospital), Shenzhen 518020, China; ^2^510120 广州，广州医科大学第一附属医院肺与重症医学研究所，国家呼吸疾病临床研究中心，国家呼吸医学中心，呼吸疾病国家重点实验室; ^2^Pulmonary and Critical Care Medicine, Guangzhou Institute of Respiratory Health, National Clinical Research Center for Respiratory Disease, National Center for Respiratory Medicine, State Key Laboratory of Respiratory Diseases, The First Affiliated Hospital of Guangzhou Medical University, Guangzhou 510120, China

**Keywords:** 新抗原, 白细胞介素7, 趋化因子19, 肺肿瘤, 新抗原反应性T细胞, Neoantigens, IL-7, CCL19, Lung neoplasms, NRT

## Abstract

**背景与目的:**

新抗原反应性T细胞（neoantigen reactive T cell, NRT）具有抑制表达特异性新抗原的肿瘤生长的能力。然而，由于免疫浸润困难和肿瘤微环境的抑制，NRT在实体瘤中的治疗效果有限。本研究针对小鼠肺癌细胞设计出可以同时表达白细胞介素7（interleukin 7, IL-7）和趋化因子19（chemokine C-C motif ligand 19, CCL19）的NRT细胞（7×19 NRT），并对7×19 NRT细胞和常规NRT细胞的抗肿瘤效果差异进行了评估。

**方法:**

针对小鼠Lewis肺癌细胞（Lewis lung carcinoma, LLC）进行了新一代测序和新抗原预测，制备了RNA疫苗，培养了NRT细胞，构建了编码IL-7和CCL19的逆转录病毒载体，转导NRT细胞并成功表达IL-7和CCL19，成功获得了7×19 NRT，并在小鼠体内外对其抗肿瘤效果进行了评估。

**结果:**

7×19 NRT细胞通过分泌IL-7和CCL19显著增强T细胞的增殖和侵袭能力，在小鼠肺癌中实现了显著的抑瘤作用，延长了小鼠的生存期。经7×19 NRT治疗后，T细胞浸润肿瘤组织及肿瘤组织坏死显著增加。此外，7×19 NRT治疗与常规NRT治疗均安全。

**结论:**

通过IL-7和CCL19的表达，NRT细胞的抗实体瘤能力显著增强，这是一种对NRT安全有效的基因修饰。

## Abstract (en)

**Background and objective:**

Neoantigen reactive T cell (NRT) has the ability to inhibit the growth of tumors expressing specific neoantigens. However, due to the difficult immune infiltration and the inhibition of tumor microenvironment, the therapeutic effect of NRT in solid tumors is limited. In this study, we designed NRT cells (7×19 NRT) that can express both interleukin-7 (IL-7) and chemokine C-C motif ligand 19 (CCL19) in mouse lung cancer cells, and evaluated the difference in anti-tumor effect between 7×19 NRT cells and conventional NRT cells.

**Methods:**

We performed next-generation sequencing and neoantigen prediction for mouse Lewis lung carcinoma (LLC), prepared RNA vaccine, cultured NRT cells, constructed retroviral vectors encoding IL-7 and CCL19, transduced NRT cells and IL-7 and CCL19 were successfully expressed, and 7×19 NRT was successfully obtained. The anti-tumor effect was evaluated in vivo and in vitro in mice.

**Results:**

The 7×19 NRT cells significantly enhanced the proliferation and invasion ability of T cells by secreting IL-7 and CCL19, achieved significant tumor inhibition in the mouse lung cancer and extended the survival period of mice. The T cell infiltration into tumor tissue and the necrosis of tumor tissue increased significantly after 7×19 NRT treatment. In addition, both 7×19 NRT treatment and conventional NRT treatment were safe.

**Conclusion:**

The anti-solid tumor ability of NRT cells is significantly enhanced by the arming of IL-7 and CCL19, which is a safe and effective genetic modification of NRT.

肺癌正在威胁着人类的健康和生命，近25%的癌症死亡与肺癌有关^[1]^。对于癌症晚期的患者，免疫检查点抑制剂（immune checkpoint inhibitors, ICIs）和靶向治疗药物虽然能够显著改善部分患者的预后^[2,3]^，但最终大多数患者会产生耐药性或者不良反应^[4]^，最终只有20%-30%的肺癌患者获益^[3]^。因此，迫切需要有效、低毒的治疗方法来提高肺癌患者的生存期和生活质量。

由于美国食品药品监督管理局（Food and Drug Adminis-tration, FDA）批准了三种靶向CD19的嵌合抗原受体T细胞（chimeric antigen receptor T cells, CAR-T）产品，过继细胞疗法（adoptive cell therapy, ACT）掀起了热潮^[5]^。目前在肺癌治疗中已经开展了多种CAR-T细胞疗法的临床试验。其中一项对肺癌进行的以表皮生长因子受体（epidermal growth factor receptor, EGFR）为靶点的CAR-T治疗（NCT01869166）^[6]^无明显毒副反应，2例患者达到部分缓解，5例患者2-8个月病情稳定。另一项I期试验（NCT03182816）^[7]^也证实了CAR-T细胞治疗肺癌患者的安全性和可行性。弗雷德哈钦森癌症研究所进行的一项CAR-T细胞临床研究（NCT02706392）^[8]^用于治疗晚期受体酪氨酸激酶样孤儿受体1（receptor tyrosine kinase like orphan receptor 1, ROR1）阳性及分期IV期的非小细胞肺癌和三阴性乳腺癌，招募的30例患者中至少有6例没有剂量限制性毒性。此外，目前CAR-T细胞治疗肺癌的多项临床研究正在进行中（NCT02414269, NCT02580747）^[9]^。然而，使用CAR-T细胞疗法治疗肺癌仍处于早期探索的阶段。

越来越多的证据^[10]^表明，来自肿瘤特异性体细胞突变的新抗原（neoantigens）正成为ACT最有吸引力的靶点。既往的研究^[11,12]^表明，患者的血液和肿瘤组织中存在许多新抗原反应性T细胞（neoantigen reactive T cells, NRT）。越来越多的临床试验^[13]^表明，转移性胆管癌、转移性乳腺癌、结直肠癌和集管癌患者在给予NRT治疗后均实现了客观缓解。研究^[14]^发现，在小鼠肺癌中过继转移NRT细胞治疗后具有良好的抗肿瘤作用。此外，一项使用个性化新抗原肽脉冲自体树突状细胞（dendritic cell, DC）疫苗治疗肺癌的研究^[15]^发现，晚期肺癌患者的客观缓解率（objective response rate, ORR）为25%，疾病控制率（disease control rate, DCR）为75%。

然而，上述的研究结果只是针对一些病例报告，在大量患者中采用NRT治疗的效果仍令人失望。输送不良、持久性不足和T细胞抑制活性有限等障碍也导致了ACT治疗在实体瘤中无法实现理想的疗效^[16]^。为了提高ACT治疗的抗肿瘤效果，研究者们尝试了多种方法。例如，放化疗和ICIs联合可提高ACT在实体瘤的治疗效果^[17]^。此外，有研究^[18]^通过额外的遗传修饰，T细胞可诱导或组成性地分泌活性细胞因子或表达配体，提高实体肿瘤的疗效和持久性。

在这些细胞因子中，白细胞介素-7（interleukin 7, IL-7）和趋化因子19（chemokine C-C motif ligand 19, CCL19）是两个非常好的选择。研究^[19]^表明，来自于外周的T细胞和DC是通过在淋巴器官中T区成纤维网状细胞产生IL-7和CCL19形成和维持的。小鼠Lewis肺癌细胞（Lewis lung carcinoma, LLC）来源于雄性C57BL/6L小鼠，它包含广泛的体细胞突变，这种小鼠癌症与人类肺腺癌相似^[20]^。由于具有高突变负担的癌症通常对免疫疗法反应积极，具有高突变负担的LLC在免疫疗法的研究和开发中发挥着重要作用^[21]^。因此，本研究旨在通过输注表达IL-7和CCL19的NRT细胞将IL-7和CCL19靶向递送到肿瘤部位，以研究共表达IL-7和CCL19的NRT对小鼠肺肿瘤模型的治疗效果。

## 1 材料和方法

### 1.1 细胞系和小鼠

杭州子源实验动物科技有限公司提供了6-8周性别匹配的C57BL/6小鼠，相关的动物实验均在无病原体环境下进行完成，并通过了深圳市人民医院、中国动物实验伦理委员会的批准。来源于美国菌种保藏中心的LLC细胞系在RPMI-1640培养基中培养，培养基中添加10%胎牛血清（fetal bovine serum, FBS）、100 U/mL青霉素和100 g/mL链霉素，培养箱条件设置为37 ^o^C和5%CO_2_。

### 1.2 新一代测序和新抗原预测

测序和新抗原预测的步骤按照先前的描述进行^[22]^。根据试剂盒要求，从LLC细胞和C57BL/6小鼠尾部组织中提取DNA和RNA后，建立全外显子组和转录组文库。然后利用Illumina Novaseq 6000平台对文库进行测序。使用BWA将DNA读数与参考基因组mm10进行比较以发现突变^[23]^。然后利用匹配的肿瘤和正常样本的读数，利用MuTect2识别肿瘤样本中包括snv和indel的变异^[24]^。使用Bowtie将RNA读数与mm10参考基因组和转录组进行比较^[25]^。通过将已知基因转录本与UCSC数据库中精确的外显子位置进行匹配，然后计算出基因表达量，并将其归一化为RPKM单位^[26]^。然后使用MuPeXI预测新抗原^[22]^。最终根据其人白细胞抗原（human leukocyte antigen, HLA）分子结合亲和力、表达水平、与自身肽的相似性以及突变等位基因的频率，对每个肽进行打分。具体打分规则按如下进行排序：（1）对主要组织相容性I类分子（major histocompatibility class I molecules, MHCI）和MHCII等位基因均具有较强的结合亲和力[半抑制浓度（half maximal inhibitory concentration, IC_50_）<150 nmol/L]；（2）对MHCI或者MHCII等位基因具有较强的结合亲和力；（3）突变基因RNA水平突变频率高；（4）对HLA I类或HLA II类等位基因结合亲和力适中（150 nmol/L<IC_50_≤500 nmol/L）^[27]^。

### 1.3 RNA疫苗制备

串联微型基因（tandem mini-genes, TMGs）的构建方法如前文所述^[28]^。根据编码突变氨基酸位点为中心以野生型蛋白序列的上游和下游天然氨基酸，延伸至共27个氨基酸的基因序列，形成单个非同义替换突变微型基因。多个微型基因串联排列，不使用额外的连接序列合成[生工生物技术（上海）有限公司]TMGs。使用信号肽（signal peptide, SP）和主要组织相容性I类分子转运结构域（MHC class I trafficking domain, MITD）以优化翻译和MHC I/II类表位加工方面合成效率^[29]^。利用现有的EcoRI和BamHI切割位点，将TMGs插入pcDNA3.1载体中，即minigene-pcDNA3.1质粒。

使用限制性内切酶Fast Digest xhol对构建好的minigene-pcDNA3.1质粒进行线性化。使用Mmessage mmachine T7 Ultra Kit以约1 µg的线性化质粒为模板进行体外转录（in vitro transcription, IVT）制备RNA。然后使用RNeasy®Plus Mini Kit纯化RNA。使用纳米分光光度计测定RNA的质量和纯度，纯度要求A260:A280为1.8-2.1。将获得的RNA疫苗放入无菌无酶离心管中，保存在-80 ^o^C以备后续应用。

### 1.4 共表达IL-7和CCL19 的NRT（7×19 NRT）的制备

#### 1.4.1 NRT的制备

从LLC荷瘤的C57BL/6小鼠中制备骨髓源性DC和T细胞^[14]^。使用Amaxa®小鼠DC细胞转染试剂盒将RNA疫苗转染入DC中。电转24 h后，T细胞和DC在含有10 ng/mL IL-7的培养液中共培养，然后在72 h后补充100 U/mL IL-2（Thermo Fisher）。每隔3天，用含IL-2（100 U/mL）的新鲜培养基半量换液，并根据需要的细胞量延长培养时间。最终，获得的NRT细胞使用磷酸盐缓冲盐水（phosphate buffer saline, PBS）清洗和收集。

#### 1.4.2 共表达IL-7和CCL19逆转录病毒载体的构建

利用2A肽序列连接IL-7和CCL19，串联构建小鼠IL-7和CCL19逆转录病毒载体（武汉金开瑞生物工程有限公司）。在 15 cm培养皿中接种293T细胞，接种浓度为1.5×10^7^。第2天，用基于聚乙烯亚胺的DNA转染试剂，用表达IL-7和CCL19的逆转录病毒包装质粒pCL-Eco（Addgene）转染293T细胞。6 h后更换培养基。转染48 h后收集病毒上清液^[30]^。

#### 1.4.3 T细胞的转导

用50 ng/mL OKT3（Ortho Biotech, Horsham, PA）激活NRT细胞，2 d后收获细胞，然后在重组人纤维蛋白片段包被的24孔板上用逆转录病毒进行转导。转导后细胞在添加10%FBS和IL-2（300 U/mL）的RPMI-1640培养基中培养^[28]^。

#### 1.4.4 流式细胞术检测

采用CD3-APC和CD137-PE抗小鼠单克隆抗体（克隆：41BB，San Diego，CA）对细胞表面进行染色。LLC细胞和NRT细胞以2:1的比例共培养。8 h后收集NRT细胞，300 g离心10 min，细胞使用荧光激活细胞分选器（fluorescence activated cell sorter, FACS）缓冲液清洗。细胞和抗体在冰上孵育30 min后，细胞再次使用FACS缓冲液清洗，并使用MACSQuant流式细胞仪（Miltenyi Biotech）进行读取。使用FlowJo软件对样品进行检测分析（BD Bioscience）。

#### 1.4.5 细胞因子释放实验

将NRT细胞与肿瘤细胞共培养，24 h后取上清液。使用酶联免疫吸附试验（enzyme-linked immunosorbent assay, ELISA）试剂盒（MultiSciences Biotech）检测NRT细胞分泌的小鼠IL-7和CCL19的量。取小鼠外周血1000 g离心30 min，取小鼠血清和肿瘤组织进行细胞因子释放试验，用ELISA试剂盒测定结果。

#### 1.4.6 细胞毒性检测

将NRT细胞与靶癌细胞分别按效应靶比5:1、10:1和20:1共培养，共培养24 h后，使用CytoTox 96细胞毒性试剂盒（Promega）测量上清中乳酸脱氢酶的水平。

#### 1.4.7 细胞增殖试验

使用细胞活力检测试剂盒（cell counting kit-8, CCK-8）法检测NRT细胞的体外生长情况。将NRT细胞接种到96孔板中，用小鼠LLC刺激。在90 µL培养基中加入10 µL CCK-8。细胞在37 ^o^C下孵育2 h后收获，在450 nm处测量光密度。进行3次独立的实验。

#### 1.4.8 细胞迁移试验

使用孔径为5 µm的聚碳酸酯过滤器（康宁），通过96孔跨孔室迁移实验评估NRT细胞的趋化性。在下层室刺激NRT细胞后，用CytoTell Blue预先标记的应答细胞在上层室孵育6或12 h。利用流式细胞术观察细胞从上腔室迁移到下腔室的情况。

### 1.5 小鼠体内的抗肿瘤研究

#### 1.5.1 小鼠肺模型的过继细胞治疗

将5×10^5^ LLC细胞植入6-12周龄的C57BL/6小鼠两侧。肿瘤接种7 d后，静脉给药100 mg/kg环磷酰胺（Cytoxan, CTX）。第1天通过尾静脉给荷瘤动物回输NRT细胞。每隔2到3天，测量小鼠的体重和肿瘤大小。当肿瘤体积达到1500 mm^3^时处死小鼠。所有动物实验均经深圳市人民医院机构动物护理与使用委员会批准。

#### 1.5.2 组织病理学评价

使用3.5%甲醛固定切除的肿瘤组织，进行苏木精-伊红（hematoxylin and eosin, H&E）染色（中国深圳生物病理研究所有限公司）。进行免疫荧光的样本，立即将切除的肿瘤组织冷冻在最佳切削温度化合物（optimum cutting temperature compound, OCT）块中。切成5 µm厚度的冷冻切片并附着在生理盐水包被的载玻片上。冷冻切片在室温下用4%多聚甲醛固定30 min，然后用阻断液（1%牛血清白蛋白，2%驴血清，0.1 mol/L PBS）在室温下阻断30 min。使用CD137一抗在4 ^o^C下染色过夜，二抗在室温下染色1 h。采用Hoechst 33342（赛默飞世尔科技）进行核染色。使用自动显微镜对染色切片进行观察拍照。在每个高倍视野（×100）评估CD137^+^细胞的数量。从每个组织块的4个连续切片中随机选择14个显微镜视野，测量CD137^+^细胞的数量。

### 1.6 统计分析

采用GraphPad软件8.0.1进行统计分析，采用双尾Student 's t检验来检测均值之间的差异，P<0.05为差异有统计学意义。

## 2 结果

### 2.1 新抗原预测和NRT的制备

提取DNA后，对LLC细胞和C57BL/6小鼠尾部组织进行全外显子组测序，并检测产生的基因突变。此外，收集RNA，并进行转录组测序以评估基因表达量。使用MuPeXI方法分析新抗原，最后选择得分最高的10个新抗原（表1）。

**表1:** 选取的新抗原信息

Augmented_peptide	Gene_Symbol	HLA_Allele	Amino_Acid_Change	Priority _Score
TMTSSQSMNFSLMSTSTVGLGLPMSRSQNTD	Clint1	H-2-Db	N/S	100
VYEAPGFQGQSWEVSGDIYNLQQPEDSQSPQ	Crybg2	H-2-Kb	R/G	100
VDGVVYLKELEPVNTTIAFFTIRDPEGKYKI	Pcdh20	H-2-Kb	P/T	100
DTLARDEFNLQKMMVMVTASGKLFGIESSSG	Emc1	H-2-Kb	T/M	100
LLPHVLVPSCPPLTRRLRLFPLHLMKPLVVF	Cmpk1	H-2-Kb	T/R	100
PFEPYISMDAMPGVFYHRNGAGLVAPVLTIQ	Nipal3	H-2-IAb	D/Y	99
APVLAAPAVAPGQVSAIDTSPASPSMPQTTL	C77080	H-2-IAb	T/A	89
PSLISTTPASSSSSNASSPSPSDTSSHKKQR	Smtn	H-2-IAb	S/A	88
YEYQGKKQPAMLRVTSFQVANSKVNATMIDH	Csmd2	H-2-IAb	G/S	79
FGEKALQGEDVRTANAIAAEAVTCLVIDRDS	Prkg1	H-2-IAb	V/A	64

HLA: human leukocyte antigen.

### 2.2 共表达IL-7和CCL19的NRT（7×19 NRT）的制备

为了获得NRT细胞将编码这10条新抗原氨基酸的基因融合在一起制备TMGs。然后将TMG用作模板来制备IVT RNA（图1A）。为了构建minigene-pcDNA3.1质粒，将TMG插入pcDNA3.1载体中。然后利用这些质粒产生IVT RNA。之后，使用上述技术转染DC细胞。采用定量聚合酶链式反应（polymerase chain reaction, PCR）检测DC细胞的转染效率，结果显示，与常规DC细胞相比，Neo-DCs中的新抗原IVT RNA显著增加（图1B）。所以，在本次实验中Neo-DCs的制备是可行的。使用上述技术收集Lewis荷瘤小鼠的T细胞，使用流式细胞术检测CD3^+^CD137^+ ^T细胞百分比，以评估与Neo-DCs共培养48 h后NRT细胞的数量。T细胞与其靶细胞之间的特定关系由共刺激标记物CD137标记，CD137在活化的T细胞上表达^[31]^。正如预期的那样，CD3^+^CD137^+^ T细胞的比例从常规T细胞中的0.15%上升到NRT细胞中的22.08%（图1C、1D），表明NRT细胞在整体T细胞回输中的比例有所上升，并具有潜在的抗肿瘤作用。

**图1:**
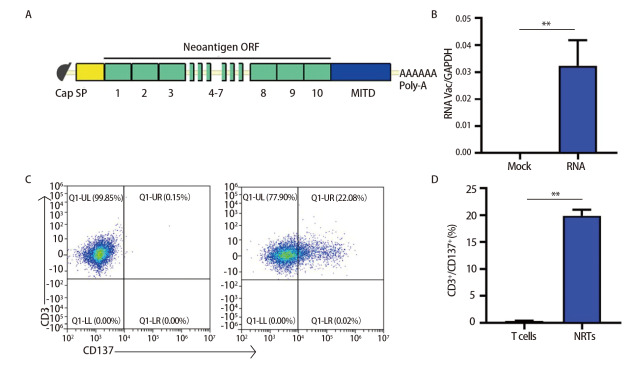
NRT的制备。A：串联微基因RNA疫苗的基本结构，10个新抗原微基因融合在一起构成串联微基因；B：从RNA转染的DC细胞（RNA）和非RNA转染的DC细胞（Mock）中提取总RNA。采用定量PCR法检测体外转录RNA的表达。数据归一化为GAPDH表达。所示数据是三个独立实验中的一个；C、D：收集T细胞前或与DC共培养48 h（NRTs）后。流式细胞术分析活化T细胞（NRT）（CD3^+^/CD137^+^）细胞群百分比。**P<0.01。

为了获得表达IL-7和CCL19的NRT细胞（7×19 NRT），用2A肽序列构建了编码IL-7和CCL19的串联构建体。本研究中使用的逆转录病毒载体结构示意图如图2A所示。逆转录病毒转导NRT细胞表达IL-7和CCL19，未转导的NRT细胞作为对照。7×19 NRT细胞与LLC细胞共培养时产生IL-7和CCL19（图2B）。

**图2:**
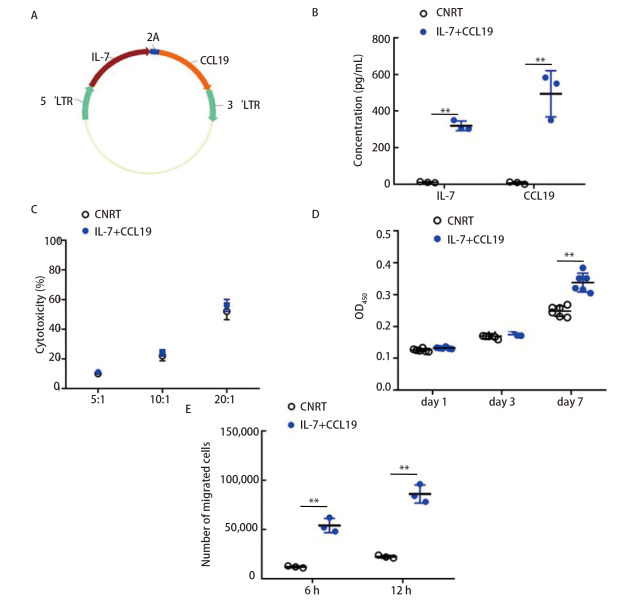
共表达IL-7和CCL19的NRT（7×19 NRT）改善了免疫功能。A：逆转录病毒IL-7和CCL19（7×19）基因结构示意图；B：7×19 NRT细胞（IL-7+CCL19）和常规NRT细胞（CNRT）与LLC细胞共培养3 d。ELISA法检测培养细胞上清液中IL-7或CCL19的浓度；C：以LLC细胞为靶点的细胞毒性试验。靶细胞与常规或7×19 NRT细胞按指定的效应/靶（E/T）比例混合；D：常规或7×19 NRT细胞与LLC细胞共培养，CCK-8分析不同天数NRT细胞的生长情况；E：常规或7×19 NRT细胞与LLC细胞共培养。第3天在上腔中加入CytoTell Blue染色的T细胞，孵育6或12 h，流式细胞术检测细胞从上腔迁移到下腔的情况。所示数据是三个独立实验的代表。**P<0.01。

由于已知IL-7可以增强T细胞的增殖和存活^[32]^，我们研究了NRT细胞的绝对数量。与传统的NRT细胞相比，7×19 NRT细胞的数量要高得多（图2C）。由于CCL19是T细胞和DC细胞的化学引诱剂^[33]^，我们接下来进行了跨井迁移试验。与传统的NRT细胞相比，与7×19 NRT细胞孵育后，应答T细胞的迁移能力显著增强（图2D）。细胞毒性实验数据亦显示常规和7×19 NRT细胞对LLC细胞具有相同的溶瘤潜能（图2E）。

### 2.3 过继7×19 NRT细胞治疗小鼠LLC肺肿瘤模型

为了探讨7×19 NRT细胞在体内的抗肿瘤作用，我们用LLC细胞接种C57BL/6小鼠两侧。当肿瘤生长到100 mm^3^时，CTX预处理后回输7×19 NRT细胞（图3A）。7×19 NRT细胞回输后，小鼠的肿瘤生长速度较PBS、CTX和未转导的NRT细胞组的肿瘤生长速度慢（图3B、3C）。作为动物整体健康的替代指标测量了体重，各组动物的体重没有明显变化（图3D）。此外，与对照组相比，7×19 NRT细胞治疗后的荷瘤小鼠存活时间延长（P<0.05，图3E）。

**图3:**
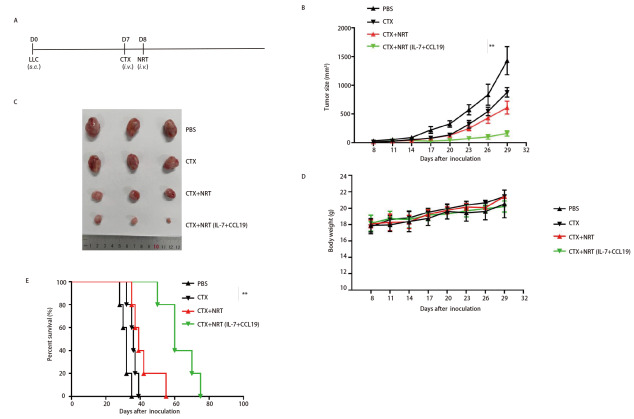
共表达IL-7和CCL19的NRT（7×19 NRT）增强了小鼠对LLC肿瘤的抗肿瘤免疫。A：体内抗肿瘤实验方案。第0天，将5×10^5^ LLC细胞皮下注射到6周龄雌性C57BL/6小鼠体内；第7天，小鼠静脉注射CTX（100 mg/kg）；第8天，小鼠静脉注射1×10^7^ NRT细胞；B、C：每3天观察肿瘤生长情况，记录肿瘤平均大小（mm^3^）；D：每3天测量各组体重；E：Kaplan-Meier生存曲线所示。**P<0.01。

### 2.4 7×19 NRT细胞可显著增加T细胞浸润和肿瘤坏死

进一步测定了血清和肿瘤中IL-7和CCL19的含量，结果显示在7×19 NRT细胞治疗的小鼠中，IL-7和CCL19均升高，而在PBS、CTX和未转导的NRT细胞组的小鼠中几乎检测不到（图4A、4B）。结果还表明肿瘤中IL-7和CCL19的浓度远高于血液中的浓度，这也显示了我们设计在肿瘤部位诱导高水平IL-7和CCL19的优势。既往研究^[34]^发现，肿瘤部位存在IL-7和CCL19可促进T细胞浸润，可增加肿瘤坏死，对生存有积极作用。注射NRT细胞8 d后观察了肿瘤组织T细胞的浸润情况，与PBS、CTX和未转导的NRT细胞相比，7×19 NRT细胞组肿瘤中浸润的T细胞较多（图4C）。在7×19 NRT细胞治疗的小鼠中观察到肿瘤坏死区域，而在常规NRT细胞治疗的小鼠中没有观察到（图4D）。

**图4:**
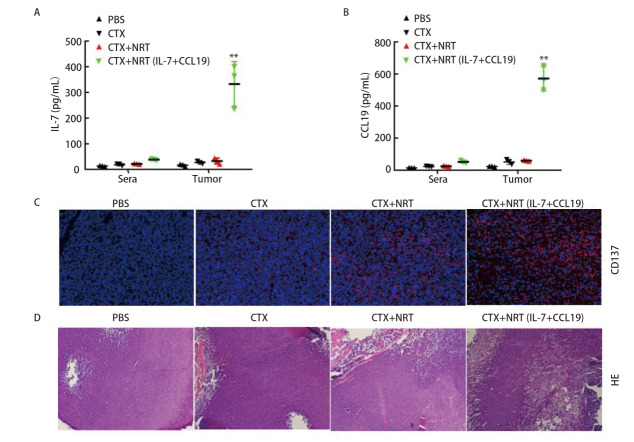
共表达IL-7和CCL19的NRT（7×19 NRT）表达IL-7和CCL19显著增加T细胞浸润和肿瘤坏死。A、B：采用ELISA法测定治疗后8天小鼠血清及肿瘤中IL-7（A）、CCL19（B）的含量。所示数据是三个独立实验的代表；C：用免疫荧光法检测治疗后8天肿瘤T细胞浸润情况；D：HE染色检测治疗后第8天肿瘤坏死情况。显微镜检查HE和IHC样品进行×100倍放大。**P<0.01。

研究结果表明，体内回输7×19 NRT细胞可以诱导分泌IL-7和CCL19，增加T细胞浸润，增加肿瘤坏死，从而增强抗肿瘤免疫。

## 3 讨论

本文构建了一个IL-7和CCL19逆转录病毒表达载体来修饰NRT细胞。体外和体内实验结果表明，IL-7和CCL19新型NRT细胞在小鼠肺肿瘤模型中具有较强的抗肿瘤作用。

对于肺癌的治疗，已经进行了一些临床试验来测试不同的细胞治疗策略，包括CAR-T细胞、T细胞受体（T cell receptor, TCR）T细胞、细胞因子诱导杀伤细胞（cytokines induce killer cells, CIK）、肿瘤浸润淋巴细胞（tumor infiltrating lymphocyte, TIL），尽管我们在努力改善T细胞的功能，但在肺癌方面的效果依旧不太理想，肺癌缺乏理想的T细胞治疗靶点是一个重要原因^[35]^。靶向“新抗原”（仅在肿瘤细胞中表达的体细胞突变）可杀死肿瘤，而不会对重要的健康组织造成不当损害。因此，过继性输注NRT成为肿瘤突变负荷高的肺癌的理想治疗方法。然而，在我们之前的肺癌小鼠研究中，利用NRT的治疗效果有限，未来的工作需要进一步提高NRT的疗效。Eric Tran等^[28]^发现，增加NRT细胞数量可显著提高其治疗效果。然而，细胞数量的增加可能会显著增加临床使用的成本。CAR-T细胞经工程改造后可分泌其他细胞因子或趋化因子，在实体瘤中表现出了增强的活性和功能。这种基因工程改造使CAR-T细胞能够分泌包括IL-7、IL-12、IL-15、IL-18、IL-23、CCL19和CCL21等细胞因子和趋化因子^[36]^。在小鼠实验中，与传统CAR-T细胞相比，IL-7和CCL19修饰的CAR-T细胞实现了对实体瘤的完全消退，延长了小鼠的生存期，具有更强的抗肿瘤活性^[34]^。除了CAR-T以外，IL-7和CCL19也被用于修饰更多的其他T细胞。Yoshihiro Tokunaga等^[37]^证实同时产生IL-7和CCL19的P1A肿瘤抗原特异性TCR-T细胞可与程序性死亡受体-1（programmed cell death protein 1, PD-1）联用产生了有效且持久的抗肿瘤免疫。然而，这种CAR-T改良策略能否应用于提高NRT细胞的治疗效果还有待进一步探索。我们的研究结果证实了针对小鼠肺癌设计出的共表达IL-7和CCL19 NRT细胞对较大肿瘤具有很强的抗肿瘤作用。分泌IL-7和CCL19可促进NRT细胞浸润，增加肿瘤坏死，进一步增强抗肿瘤免疫。

研究^[28]^表明，肺癌等突变负荷高的肿瘤非常适合免疫治疗。然而，高突变负担往往会导致免疫逃逸和复杂的肿瘤微环境（tumor microenvironment, TME）^[38]^。TME具有深度免疫抑制作用，这也是大多数通过刺激免疫细胞对抗癌症的癌症疗法临床疗效有限的关键原因。肿瘤细胞外基质的形成和趋化因子分泌的减少抑制了免疫细胞的浸润。TME中存在着肿瘤相关巨噬细胞（tumor associated macrophage, TAM）、骨髓来源的抑制性细胞（bone marrow derived suppressor cells, MDSCs）、调节性T细胞（regulatory T cell, Treg）等免疫抑制细胞，以及IL-10、转化生长因子-β（transforming growth factor-beta, TGF-β）、吲哚胺2,3-双加氧酶（indoleamine 2,3-dioxygenase, IDO）等抑制性细胞因子，都会显著抑制浸润免疫细胞的功能，导致肿瘤细胞缺乏杀伤作用^[39]^。因此，如何克服这些抑制剂对细胞免疫治疗尤为重要。既往研究^[14]^证实，新抗原诱导的NRT细胞具有肿瘤识别和杀伤作用，但在小鼠体内的抗肿瘤作用有限，主要原因是肿瘤浸润不足，NRT细胞活力有限。在本研究中，我们用经典的细胞因子IL-7和CCL19来修饰NRT细胞，并评估其抗肿瘤性能。在小鼠模型中修饰的NRT细胞具有更强的肿瘤侵袭能力和增殖活性，更能杀伤已构建的大肿瘤。这项研究的结果表明，对于具有复杂免疫微环境的高度突变的肿瘤，如肺癌，过继性回输修饰的NRT细胞是一种理想的治疗方法。

然而，本研究依然有一些缺陷，没有探索7×19 NRT细胞用于过继性转移治疗的数量，需要进一步的研究来确定多少修饰的NRT细胞可能引起细胞毒性，以及多少修饰NRT细胞可以达到预期的治疗效果；其次，肿瘤模型数量也很少，而且只在啮齿动物肺癌上进行过测试，需要进一步的研究来确定类似的结果是否适用于其他肿瘤。

综上所述，本项研究证实NRT细胞诱导IL-7和CCL19表达增强了NRT细胞对肺癌的抗肿瘤作用，是一种安全的修饰方式，研究结果为肺癌的免疫治疗提供了一种有效和安全的策略。
